# Analysis of the mRNA Expression of Chemotherapy-Related Genes in Colorectal Carcinoma Using the Danenberg Tumor Profile Method

**DOI:** 10.1155/2013/386906

**Published:** 2013-03-16

**Authors:** Shin Sasaki, Toshiyuki Watanabe, Hiroshi Nakayama

**Affiliations:** Department of Surgery, Omori Red Cross Hospital, 4-30-1, Chuo, Ota-ku, Tokyo 143-8527, Japan

## Abstract

The establishment of individualized chemotherapy for colorectal carcinoma based on the expression of genes involved in chemotherapeutic sensitivity or prognosis is necessary. To achieve this, the expression profiles of genes within tumors and their relationship to clinicopathological factors must be elucidated. Here, we selected 10 genes (*TS, DPD, TP, FPGS, GGH, DHFR, ERCC1, TOPO-1, VEGF*, and *EGFR*), examined differences in their mRNA expression between the upper and lower thirds of tumors by laser-captured microdissection and real-time RT-PCR (the Danenberg tumor profile), and analyzed the relationships between their expression profiles and clinicopathological factors. Interestingly, the mRNA expression of *DPD, TP*, and *VEGF* was significantly higher in the lower third than in the upper third of tumors (*P* = 0.044, 0.023, and 0.013, resp.). Furthermore, increased *ERCC1* mRNA expression in the lower third of tumors correlated with recurrence (*P* = 0.049), and *VEGF* mRNA expression was significantly higher in cases with recurrence than in cases without recurrence, both in the upper and lower thirds of tumors (*P* = 0.018 and 0.036, resp.). These results implied that heterogeneity in *DPD, TP*, and *VEGF* expression may exist in colorectal carcinoma and that *ERCC-1* and *VEGF* may be markers predicting recurrence after curative operation.

## 1. Introduction

5-Fluorouracil (5-FU) and its relatives are mainstays in the chemotherapeutic treatment of colorectal carcinoma [1, 2]. Recently, several newly discovered drugs, including molecular targeted agents, have facilitated the progression and diversifications of chemotherapies. Furthermore, many studies have reported that a variety of candidates can be used to predict chemotherapeutic sensitivity or prognosis [3], and the establishment of individualized chemotherapy based on the expression profiles of these genes is necessary for promoting the efficacy of chemotherapeutic agents in both nonresponders and responders. To achieve this, differences in gene expression profiles and distributions throughout the tumor must be analyzed, and relationships between the distribution and extent of gene expression and clinicopathological factors must be elucidated. Among the many candidates reported thus far, we selected 10 genes that have been extensively analyzed as possible factors related to chemosensitivity and/or prognosis in colorectal carcinoma. Six genes (thymidylate synthetase *TS*, dihydropyrimidine dehydrogenase *DPD*, thymidine phosphorylase *TP*, folylpolyglutamate synthetase *FPGS*, gamma-glutamyl hydrolase *GGH*, and dihydrofolate reductase *DHFR*) are known to be involved in the 5-FU metabolic pathway. Excision repair complementing-1 (*ERCC-1*) and topoisomerase 1 (*TOPO-1*) are important biomarkers that predict tumor responses to oxaliplatin and CPT-11, respectively, and the 2 remaining genes (vascular endothelial growth factor *VEGF* and epidermal growth factor receptor *EGFR*) are target factors for molecular targeted agents. 

TS is the rate-limiting enzyme in *de novo* pyrimidine biosynthesis and is inhibited by 5-fluoro-2′-deoxyuridine-5′-monophosphate (FdUMP), a compound that is derived from 5-FU and facilitates the inhibition of DNA synthesis [4, 5]. DPD is the initial enzyme in the metabolic pathway responsible for the catabolism of the pyrimidine bases uracil and thymidine [6]. More than 80% of administered 5-FU is degraded by DPD* in vivo*. Many reports have demonstrated an association between TS and/or DPD expression and sensitivity to 5-FU. Moreover, individualized chemotherapy has been proposed according to tumor classification by high or low expression of TS and DPD. In advanced colorectal carcinoma, only cases with low TS and low DPD expression respond to 5-FU [7, 8]. Furthermore, *DPD* gene copy number, which correlates with *DPD* mRNA expression, is a predictor for the sensitivity of cancer patients to 5-FU-related drugs [9]. TP is a 5-FU metabolic enzyme and is also known as platelet-derived endothelial cell growth factor (PD-ECGF). In advanced colorectal carcinoma, low expression levels of both TS and TP in tumors predict very high response rates to 5-FU as well as significantly longer survival times [10]. DHFR catalyzes the reduction of dihydrofolate to tetrahydrofolate (THF). THF is required for the activity of folate-dependent enzymes and is thus essential for DNA synthesis and methylation. DHFR is a target of the folate antagonist methotrexate, and DHFR expression can affect chemosensitivity [11]. ERCC-1 is a biomarker that can be used to predict survival in colorectal cancer patients receiving combination oxaliplatin and fluorouracil [12]. 

VEGF is one of the most important signaling proteins involved vasculogenesis and angiogenesis [13, 14]. Bevacizumab is a humanized monoclonal antibody developed against VEGF and has been used as a chemotherapeutic agent in the clinic [15, 16]. However, VEGF is considered a poor prognostic factor in colorectal carcinoma [17, 18], and some studies have also demonstrated that VEGF may be involved in determining the patient's sensitivity to 5-FU [19, 20]. EGFR, a transmembrane glycoprotein, is a member of the ERBB receptor tyrosine kinase superfamily. EGFR binds to its cognate ligand EGF, which further induces tyrosine phosphorylation and receptor dimerization with other family members, leading to enhancement of uncontrolled proliferation. The proportion of cases with high EGFR expression increases according to the stage of the cancer, and high EGFR expression is related to poor prognoses in some types of cancers [21]. Monoclonal antibodies for EGFR, including cetuximab and panitumumab, have been developed for the treatment of multiple cancer types.

In the present study, we examined differences in the mRNA expression of these genes between the upper and lower thirds of colorectal tumors and analyzed the relationships between their expression profiles and clinicopathological factors. 

## 2. Materials and Methods

### 2.1. Patient Eligibility

We studied 20 patients (13 men and 7 women; mean age 67.3 years) who underwent curative operation for colorectal carcinoma with depth of muscularis propria (mp) or more between December 2005 and April 2009, at the Department of Surgery, Omori Red Cross Hospital. This study was approved by the Ethical Committee of this hospital. All patients were informed of the nature of this study, and written informed consent was obtained. 

### 2.2. Laser-Capture Microdissection and Real-Time RT-PCR (the Danenberg Tumor Profile Method)

**Figure 1:**
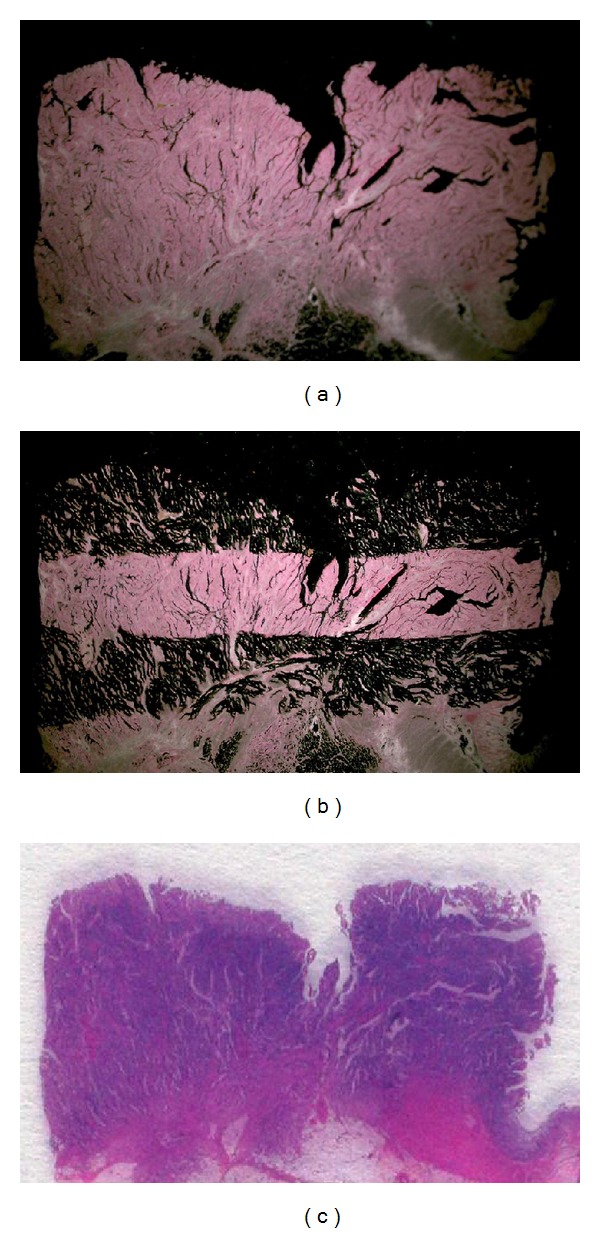
A representative preparation of samples. (a) Staining with nuclear fast red. (b) Laser-capture microdissection from the upper and lower thirds of tumors, separately. (c) Staining with H and E for histological examination.

In every case, 4 sets of 10 *μ*m sections and 1 set of 5 *μ*m sections were prepared from formalin-fixed, paraffin-embedded tissues and mounted onto slides. The latter sections were stained with H and E and examined histologically. The former sections were stained with nuclear fast red (American MasterTech Scientific, Lodi, CA) and were used for laser-capture microdissection (PALM Microsystem, Leica, Wetzlar, Germany) from the upper and lower thirds of tumors, separately (Figure 1). The dissected tissue samples were transferred to reaction tubes containing 400 *μ*L RNA lysis buffer. The samples were homogenized and heated at 92°C for 30 min, and 50 *μ*L of 2 M sodium acetate was then added at pH 4.0, followed by 600 *μ*L phenol/chloroform/isoamyl alcohol (250 : 50 : 1). The tubes were placed on ice for 15 min and then centrifuged at 13000 rpm for 8 min in a chilled centrifuge. The upper aqueous phase was carefully removed. Ten microliters glycogen and 300–400 *μ*L isopropanol were added. The samples were chilled at −20°C for 30–45 min to precipitate the RNA and were then washed in 500 *μ*L of 75% ethanol and air-dried for 15 min. The pellet was resuspended in 50 *μ*L of 5 mM Tris. Finally, cDNA was prepared as described by Lord et al. [22]. 

Quantification of 10 genes of interest (*TS*, *DPD*, *TP*, *FPGS*, *GGH*, *DHFR*, *ERCC-1*, *TOPO-1*, *VEGF*, and *EGFR*) and an internal reference gene (*β*-actin) was performed with a fluorescence-based real-time PCR system (ABI PRISM 7900 Sequence Detection System, TaqMan, Perkin-Elmer Applied Biosystems, Foster City, CA). The PCR reaction mixture consisted of 1200 nM of each primer, 200 nM probe, 0.4 U AmpliTaq gold polymerase, 200 nM each of dATP, dCTP, dGTP, and dTTP, 3.5 mM MgCl_2_, and 1 × Taqman buffer A containing a reference dye. The final volume of the reaction mixture was 20 *μ*L. Cycling conditions and the primers and probes were described previously by Matsubara et al. [23]. Gene expression values (relative mRNA levels) were expressed as the ratio (difference between Ct values) between the gene of interest and an internal reference gene (*β*-actin).

Four samples were chosen at random, and another set of sections was prepared from these formalin-fixed, paraffin-embedded tissues. Gene expression values were analyzed again to confirm the reproducibility of the measurement of gene expression.

### 2.3. Statistical Analysis

The correlations between the mRNA expression of genes examined and clinicopathological parameters were evaluated by Student's *t*-test. Probability (*P*) values of less than 0.05 were considered statistically significant.

## 3. Results

The relative mRNA expression of the 10 genes examined (with *β*-actin as an internal control) by laser-capture microdissection and real-time RT-PCR is shown in Table 1. For genes other than *DPD*, *TP*, and *VEGF*, there were no differences in mRNA expression levels between the upper and lower thirds of the tumors. However, the mRNA expression of *DPD*, *TP*, and *VEGF *was significantly higher in the lower third of tumors than in the upper third (Table 1;  *P* = 0.044, 0.023, and 0.013, resp.). To confirm the accuracy and reproducibility of our gene expression measurements, we performed duplicate measurements for 4 randomly chosen samples. Relative differences in mRNA expression between the upper and lower thirds of tumors were calculated as follows:

(1) Δ  relative  mRNA  expression     =(relative  mRNA  expression  in  upper  third)  −(relative  mRNA  expression  in  lower  third).

**Figure 2:**
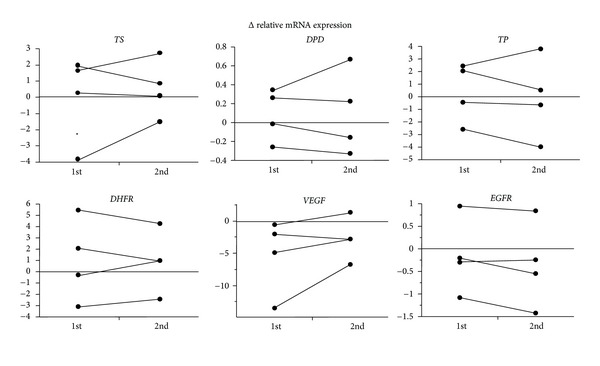
Accuracy and reproducibility of the method. The Δ relative mRNA expression of 6 genes was independently evaluated twice for 4 samples, showing the accuracy and reproducibility of the method.

**Figure 3:**
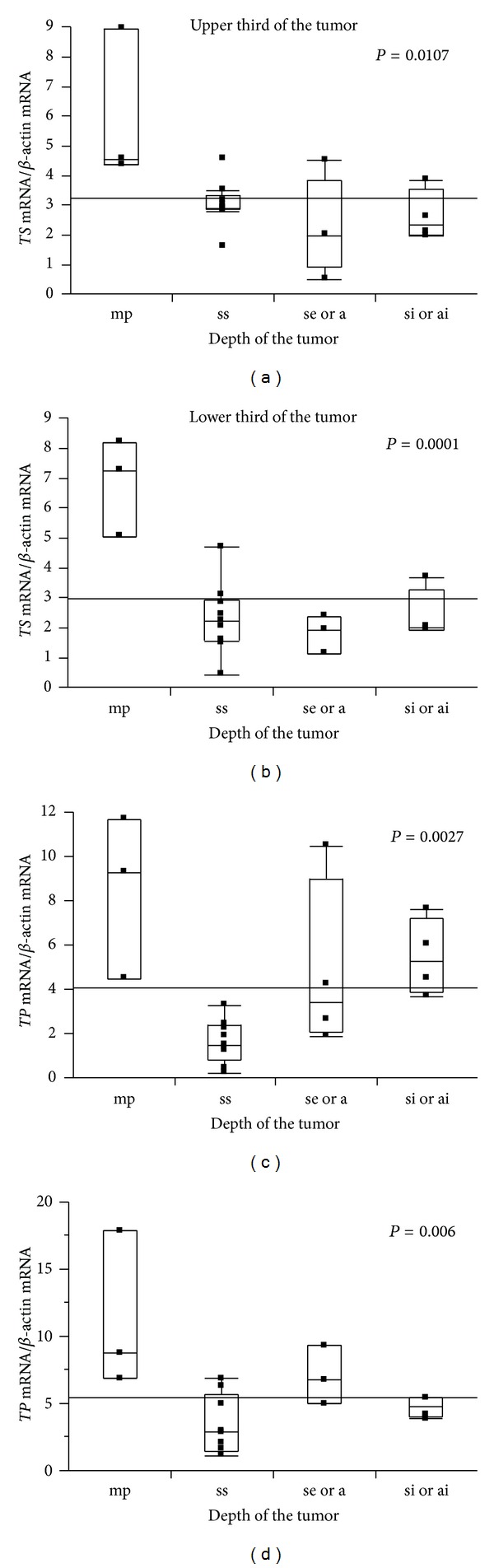
Analysis of the expression of *TS* and *TP* mRNA. *TS* and *TP* mRNA levels were significantly reduced with increasing depth of tumors in both the upper and lower thirds of tumors.

**Figure 4:**
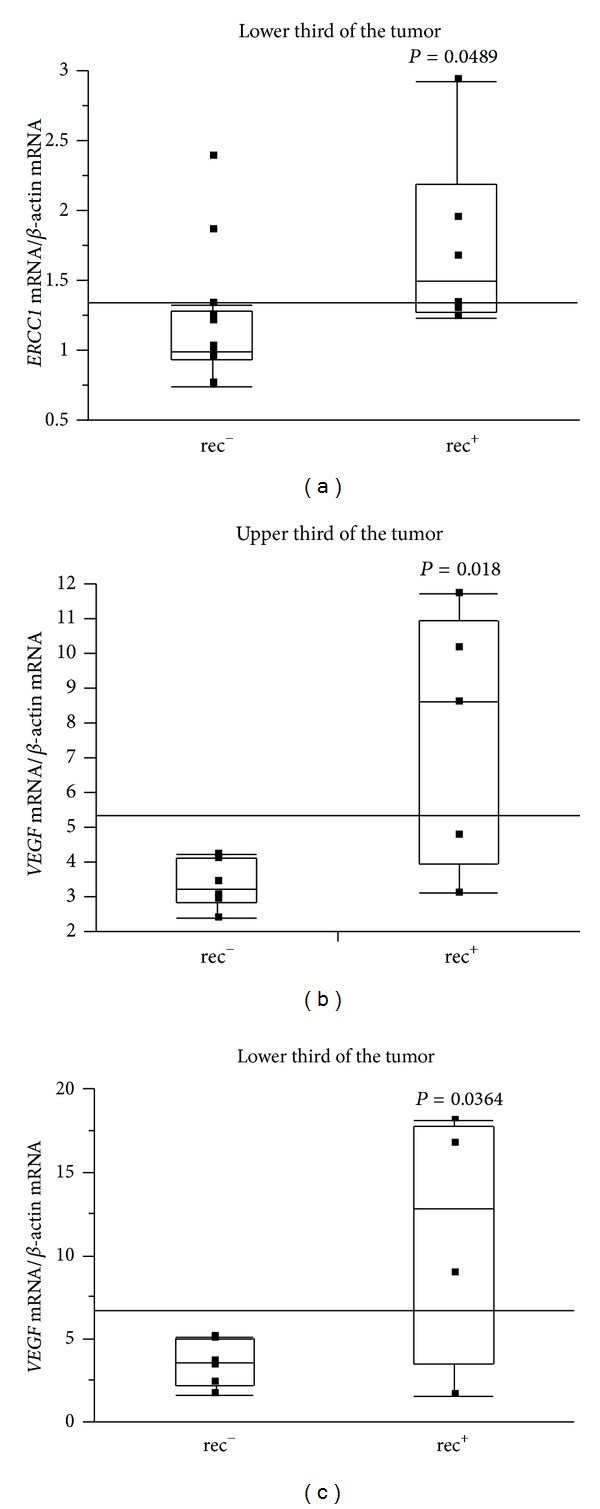
Increased expression of *ERCC-1 *mRNA in the lower third of tumors was statistically correlated with recurrence (*P* = 0.049) (a). For cases without adjuvant chemotherapy, the expression of *VEGF* mRNA was significantly higher in cases with recurrence than in cases without recurrence in both the upper third (b) and lower third (c) of tumors.

The results of Δ relative mRNA expression were almost identical for all 10 genes, as shown in Figure 2, and the accuracy and reproducibility of the method used in this study were confirmed. Also, the expression levels of *TS* and *TP* mRNAs declined as with increasing tumor depth through both sections of the tumor (Figure 3;  *P* = 0.011 and *P* = 0.003). Increased expression of *ERCC-1* mRNA in the lower third of tumors was statistically correlated with recurrence (*P* = 0.049; Figure 4(a)). Furthermore, for 11 cases given no adjuvant chemotherapy, the expression of VEGF mRNA was significantly higher in cases with recurrence than in cases without recurrence, both in the upper and in lower thirds of tumors (Figures 4(b) and 4(c); *P* = 0.018 and *P* = 0.036, resp.). Followup periods ranged from 1222 to 2272 days, with a mean followup period of 1828 days (over 5 years).

## 4. Discussion

The Danenberg tumor profile method, comprising laser-capture microdissection and real-time RT-PCR techniques, was established for the evaluation of gene expression based on extraction of mRNA from paraffin-embedded specimens. Importantly, the results were closely correlated with those from fresh-frozen sections [24], and this method has been well accepted as yielding quantitative and accurate measurement of gene expression. To confirm the accuracy and reproducibility of this method, we also evaluated the mRNA expression profiles of all genes examined a second time for 4 separate samples. As shown in Figure 2, the results were almost identical in the 2 independent evaluations, although statistical analysis was not performed because of the small number of samples. 

For the advancement of clinical therapeutics, the establishment of individualized chemotherapy based on the expression of genes related to chemosensitivity or prognosis is necessary. To achieve this, the distribution and expression of genes of interest in tumors must be elucidated. In other words, we must determine whether gene expression at the surface of tumors, where we can obtain tissue to evaluate by biopsy, would reflect the expression of the tumor as a whole. If the heterogeneity of gene expression in tumors is not clinically negligible, evaluation of gene expression in samples obtained from biopsy specimens must be deliberate. Accordingly, we focused on evaluation of gene expression in the upper and lower thirds of tumors in this study. While many reports have evaluated mRNA expression using laser-capture microdissection and real-time RT-PCR, most of these studies have used whole tumors and/or adjacent normal tissue, and no studies have evaluated the distribution of gene expression by sites in the tumor. 

The results of our analysis of the relative mRNA expression of 10 genes are summarized in Table 1. The relative mRNA expression levels of *TS* and *DPD* were 3.23/2.94 and 0.32/0.40 (in the upper third/lower third of tumors), respectively. These numbers are similar to those reported in a large-scale population analysis conducted by Fukui et al. [25], where the expression levels of *TS* and *DPD* mRNA were 1.96 and 0.34, respectively, in colorectal carcinoma; the mean expression of *TS* mRNA was slightly higher in our study. 

For genes other than *DPD*, *TP*, and *VFGF*, the differences between mRNA expression in the upper and lower thirds of tumors, defined as the Δ relative mRNA expression, were not significant. Hence, for these 7 genes (*TS*, *FPGS*, *GGH*, *DHFR*, *ERCC-1*, *TOPO-1*, and *EGFR*), evaluation of their expression by biopsy would reflect the mRNA expression in the whole tumor and would therefore permit their use in the clinic. However, the expression levels of *DPD*, *TP*, and *VEGF* mRNA were significantly higher in the lower third of tumors than in the upper third (Table 1;  *P* = 0.044, 0.023, and 0.013, resp.). This result implied that heterogeneity of *DPD*, *TP*, and *VEGF* expression may exist in colorectal carcinoma. Further investigations are required to confirm these observations.

Next, we examined the relationship between mRNA expression of genes and depth of tumor and found that the expression levels of *TS* and *TP* mRNAs declined as the depth of tumor increased in both the upper and lower thirds of tumors (Figure 3;  *P* = 0.011, *P* = 0.003). As theoretically predicted from 5-FU metabolic pathway analysis, when the expression of TS and TP increased, sensitivity to 5-FU would be reduced. Indeed, many reports have supported this hypothesis. Metzger et al. also reported that low expression levels of both TS and TP in tumors predicted a very high response rate to 5-FU and a significantly longer survival in patients with advanced colorectal carcinoma [10]. Thus, a mechanism through which hosts lower TS and TP expression to increase chemosensitivity may exist. Furthermore, for evaluation of the expression levels of these genes, depth of tumor must be taken into consideration.

Finally, we investigated the relationship between gene expression and recurrence with or without prior chemotherapy. Our data suggested that high expression of *ERCC-1* mRNA in the lower third of tumors was statistically correlated with recurrence (Figure 4(a) *P* = 0.049). Furthermore, for 11 cases given no adjuvant chemotherapy, the expression of *VEGF* mRNA was significantly higher in cases with recurrence than in cases without recurrence, both in the upper and lower thirds of tumors (Figures 4(b) and 4(c);  *P* = 0.018 and *P* = 0.036, resp.). There were no other differences in clinicopathological parameters between recurrence and nonrecurrence groups (data not shown). This result is consistent with many studies suggesting that VEGF is a poor prognostic factor for colorectal carcinoma [5, 6]. The mean followup period for cases with no adjuvant chemotherapy was 1828 days, which we considered sufficient to evaluate the relationship between gene expression and recurrence. Our results demonstrated that VEGF may be one of markers predicting recurrence after curative operations. Thus, for cases with high VEGF expression in tumors, adjuvant chemotherapy should be given.

In conclusion, we revealed that heterogeneity of *DPD*, *TP*, and *VEGF* expression may exist in colorectal carcinomas in which the depth had advanced and that ERCC-1 and VEGF may be biomarkers predicting recurrence after curative operations. Although the number of samples was small and this study was preliminary, these results must be informative and useful for the establishment of appropriate individualized chemotherapy in colorectal carcinomas.

## Figures and Tables

**Table 1:** mRNA expression profiles of 10 genes in the upper and lower thirds of colorectal tumors.

	mRNA (relative gene expression)		*P*
	Upper third of the tumor	Lower third of the tumor	
*TS *	3.23 ± 1.73	2.94 ± 2.03	N.S.
*DPD *	0.32 + 0.28	0.40 ± 0.32	0.044
*TP *	4.04 ± 3.34	5.46 ± 3.86	0.023
*FPGS *	0.53 ± 0.21	0.48 ± 0.17	N.S.
*GGH *	23.14 ± 12.72	22.72 ± 15.42	N.S.
*DHFR *	5.21 ± 2.48	4.43 ± 2.21	N.S.
*ERCC1 *	1.40 ± 0.56	1.34 ± 0.57	N.S.
*TOPO-1 *	2.66 ± 0.94	2.39 ± 0.90	N.S.
*VEGF *	6.70 ± 4.13	9.32 ± 6.19	0.013
*EGFR *	1.19 ± 0.57	1.40 ± 0.69	N.S.
